# The body as an obstacle and the “other”. How patients with chronic inflammatory bowel diseases view their body, self and the good life

**DOI:** 10.1186/s12910-024-01076-2

**Published:** 2024-07-24

**Authors:** Anke Erdmann, Christoph Rehmann-Sutter, Florian Schrinner, Claudia Bozzaro

**Affiliations:** 1 Institute for Experimental Medicine, Medical Ethics Working Group, Kiel University, Kiel, Germany; 2 Institute for History of Medicine and Science Studies, University of Lübeck, Lübeck, Germany; 3 Institute of Clinical Molecular Biology (IKMB), Genetics & Bioinformatics Research Group, Kiel University, Kiel, Germany

**Keywords:** Body image, Embodiment, Self, Inflammatory bowel disease, Quality of life, Precision medicine

## Abstract

**Background:**

Treatment of chronic inflammatory bowel disease (IBD) aims to improve patients’ quality of life and the extent of treatment success is measured via patient reported outcomes (PROs). However, questionnaires used to collect PROs often include scales that are not specific to IBDs. Improving these scales requires a deeper understanding of patients’ lived experience. With this study we give first insights and develop hypotheses on how patients with IBDs experience their body and self and how they adjust their life plans in the context of precision medicine (PM). The guiding question is to understand what they need to achieve a good life, while facing their disease.

**Methods:**

We developed a conception of the “good life” that draws on Philippa Foot’s “naturalized” approach and distinguishes six different dimensions that are relevant for a good life. This conception guided us as we conducted 10 qualitative interviews with patients suffering from IBD who were in precision medicine clinical research settings. The interviews aimed to gain insights for answering our research question: How do body experiences affect the good life of patients with IBD? We analyzed the interviews with interpretative phenomenological analysis (IPA).

**Results:**

Five group experiential themes emerged: (i) Life options and plans, (ii) other people’s responses, (iii) strategies to deal with others’ responses, (iv) perception of the body and self, and (v) perception of life as good despite suffering. We report here on three of them (i, iv and v), which are primarily relevant for evaluating the outcomes of PM care. Whereas with “life options and plans (i),” our study predominantly confirmed previous research, with “perception of the body and self (iv),” we found that some of the patients changed their relationship to their body and themselves. They perceived the body or the disease as the “other” and their self appears divorced from their own body. Although this might be an avoidance strategy patients use to assign responsibility for their condition and its “disgusting” symptoms to the “other,” it is important to include it in patient reported outcome (PRO) assessments.

**Conclusions:**

We conclude with the insight that the multi-dimensional approach based on Foot’s concept of a good life is well-suited as a basis for investigating the quality of life of people with IBD. Interviews based on this concept produced results that go beyond the understanding of health-related quality of life (HRQoL). Additionally, we offer some considerations about patients’ opportunities for achieving a good life and suggestions for further developing patient reported outcome scales.

**Supplementary Information:**

The online version contains supplementary material available at 10.1186/s12910-024-01076-2.

## Introduction

During the 20th century, chronic inflammatory bowel diseases (IBDs) such as Crohn’s disease (CD) and ulcerative colitis (UC) have been clinically observed mainly in Western countries. However, due to an adoption of Western lifestyle habits in Asia, South America, and Africa, the incidence rates are increasing globally [1]. This places an enormous burden on people and on health care systems. Without question, IBD has a significant impact on the quality of life (QoL) of patients [2, 3]. The disease impairs the ability to meet fundamental needs such as adequate nutrition, hygiene and safety. It affects psychological well-being, especially self-esteem, and makes social relationships, life activities, and participation more difficult [3]. The development of effective therapy is therefore of paramount importance to alleviate patients’ suffering. However, the therapy of IBD still resembles rather a process of trial and error, as drugs such as biologics can lead to positive effects for some patients, while causing negative side effects for others. To address this problem, researchers are currently working on multi-omics and big data approaches that are often summarized under the terms *personalized* or *precision medicine*. The aim of PM is finding a precise and effective therapy for each patient based on their individual genetic traits, lifestyle habits, and environmental exposures [4–6]. The improvements of such novel therapeutic approaches need to be evaluated scientifically and clinically. From an ethical perspective, the patient’s perspective is pertinent, usually measured by patient reported outcome scales (PROs). However, the PROs currently used only partially reflect the patients’ perspective on their actual condition. Sometimes objective criteria such as endoscopic scores are assumed to be more reliable for assessing treatment response [7]. In addition, our own study revealed that the PROs on sleep, anxiety, and stool form do not adequately capture the patient´s condition and that scales on body image and sexuality, which are important issues in IBD, are not in use [8]. Thus, there is a need for new or refined PROs which more comprehensibly and reliably represent the patients’ perspectives on their body, the experiences with the disease in their everyday life worlds and patient-reported disease activity.

This need has been the inducement for a qualitative investigation into IBD patient’s views and experiences of life with IBD in the context of PM. Which criteria can be used to establish whether a novel treatment actually makes their lives “better”? A “better” life refers to an understanding of what for these groups of patients makes human life “good.” A good life is a broad philosophical concept that has a remarkable history going back to at least Aristotle’s theory of *eudaimonia* in *Nichomachean Ethics* which focuses on virtues as key components of a good life. In order to provide our study with a theoretical framework that can accommodate patients’ embodied experiences while leading a life with their chronic disease [9], we refer to a suitable contemporary philosophical approach to the good life, that we further specify with references to phenomenology of the body.

### Foot´s concept of the good life

Foot’s approach can be described as a naturalistic and teleological conception of the good. Its basic assumption is that each species has a natural way of life [10, pp. 25–37] which to some extent determines what is good for individuals of that species. This assumption makes it possible to determine whether an individual is sick or is behaving in a dysfunctional way, (e.g. with regard to the interest of survival.) In the case of plants and animals, their natural way of life can be used as a comparative value to define their goodness. With humans, things are more complex. According to Foot, a good life for human beings involves the possibility of realizing one’s abilities and virtues, maintaining social relationships, and experiencing contentment, joy, and pleasure. This understanding of the good life goes beyond a temporary feeling of joy, pleasure, or happiness and challenges the notion that a hedonistic concept of happiness alone is the goal of the good life. Of course, pleasure and hedonistic happiness can be conducive to a good life. Yet more importantly for the topic of our paper, experiences of unpleasantness, physical pain, and long suffering from chronic illness may not always be antithetical to human flourishing.

This brings into focus the predicate of depth as a necessary condition for happiness that makes a life good. Foot explains that among different cultures, most people experience deep happiness when a child is born and deep grief when a parent passes away. Deep happiness requires seemingly ordinary things: home, family, work, and friendship. But there is also something that precludes deep happiness and that is wickedness. Foot claims that happiness bought by wickedness can never be conducive to a good life, because the person feels that it was acquired illegally. Foot understands happiness as the joy of being good, the joy one feels when pursuing and achieving the right goals [10].

Important for our study is the fact that Foot’s concept of the good life is deeply rooted in a naturalistic view of human beings. This means that she recognizes the central role that the body, with its natural functions, plays in people’s lives in achieving a good life as she sees it. Physical dysfunction and illness can be obstacles to a good life. Chronically ill people face the particular difficulty of having an illness that is incurable at the present time. This means that they have to find a way to cope with their illness, and learn to adapt to the limitations and obstacles that the disease imposes on their lives. They have to develop a new concept of themselves and of a good life that takes into account the fact that their body is not, or no longer, functioning in a way that is normal for human beings. The body is the natural, biological substratum for human existence and the medium through which human beings perceive themselves and interact with the world around them. According to Plessner, the phenomenology of the body distinguishes between “having a body” and “being a body” [11]. For Plessner, Merleau-Ponty and many other phenomenologists, our embodiment is inherently twofold: a physical object and a subjectively lived experience. Although we are constantly engaged in activities with our bodies in everyday life, we are generally not aware of the body as an object of perception. It is often our experiences of pain, disability and illness that first make us aware of the body as an object. Particularly in the context of chronic illness, the body imposes itself on perception with increasing frequency and clarity, forcing patients to come to terms with it.

Numerous factors play an important role in dealing with the sick body, including one’s own body image and the view of others. Body image can be defined as “the subjective picture of individuals of their own body.” It is a “complex construct comprising thoughts, feelings, evaluations, and behaviors related to one’s body” [12]. It is of special interest in IBD because patients suffer from symptoms that can lead to dissatisfaction with their body functions and physical appearance. These include weight loss, growth retardation, delayed onset of puberty, diarrhea, incontinence, and rectal bleeding. Medication side effects such as acne or rapid weight changes can further increase the risk of negative body image [13]. Studies show that patients with a negative body image are more likely to suffer from depression and anxiety and have suicidal thoughts. Perceiving one’s body as having a “negative appearance” can also impact relationships and quality of life [14]. In addition, Mead has shown that our relationships influence the perception of ourselves, as the self is developed, maintained or modified through social interaction with other people [15]. Zahavi (2010) as a phenomenologist claimed this as well with reference to Sartre [16] by using the example of shame. According to Sartre, shame results primarily from a person accepting how others see them and acknowledging that they are what the other thinks they are [17]. However, this recognition of the self, constructed through social interaction, is also the basis for the shaping of life and the realization of life opportunities and plans. The perception of the body and self is the central starting point from which love, friendship, and the realization of abilities and virtues in private and professional life can be attained. Mediated through the lived body we perceive the world and act in and to it, or, as Merleau-Ponty put it, the lived body is essentially a relationship to the world: “The body is the vehicle of being in the world and, for a living being, having a body means being united with a definite milieu, merging with certain projects, and being perpetually engaged therein.” [18, p. 84]. Fuchs (2007) also emphasized the significance of our social environment for life, pointing out that we determine the course of our lives by choosing our social (and spatial) environment, as this environment responds to us. The course of life develops as a circular process: on the one hand it is determined by our own activities, on the other hand by the “responses” of our social relationships [19]. For a good life, therefore, the reactions we receive from others are also decisive.

In light of this, the question is to what extent people with IBD could lead a good life in the holistic sense proposed by Foot. Living with IBD brings body experiences into the foreground. How do the specific body experience a person has with IBD and medical treatments influence what they see as a good life? These considerations motivated us to take a closer look at the connections between a changed body image in IBD, limitations of bodily functions, the resulting stigmatization, the changes in self-perception, and the influence of these experiences on life plans and the good life as a whole.

### Previous research, research question and objectives

In contrast to Foot´s broad concept of a good life and the multifaced analysis from the phenomenology of the body tradition, studies on health-related quality of life (HRQoL) focus much more narrowly on the subjective appraisal of the physical, mental, and social functioning [20, 21]. Previous qualitative studies have mostly explored specific aspects of HRQoL. These include: diet-related QoL [22–25], work- or study-related problems [26, 27], experiences with diagnostics, therapy [28] and telemedicine [29–31], patients’ priorities regarding treatment goals and measurement of treatment outcomes [32], and perceptions of body image [33].

While these publications give valuable insights into specific life areas, our concern is to explore the impact of the disease via the patients’ accounts on their good life in its entirety as far as their own assessment reaches. We believe that the perception of the own body and self, the sense of being in the world, is the foundation for many activities that enable a good life. Thus, as this factor is particularly important for assessing IBD therapy, we developed the following research question for our empirical study: **How do body experiences affect the good life of patients with IBD?**

The answer to this question is significant. It identifies what aspects and obstacles might be particularly relevant from the patients’ perspectives for them to be capable of living a good life. Our study’s main objectives are to: (1) Examine whether the holistic approach of a good life developed for example by Foot, can be used as a heuristic to gain a more comprehensive view on patients’ needs and on what is valuable in their lives as compared to conventional QoL studies; (2) Provide information for the improvement of PRO scales for patients with IBD; (3) Inform medical research and practice about the ethical evaluation of results; and (4) contribute to the question on how to personalize treatment and care in precision medicine.

## Methods

For this purpose, we conducted between August 2022 and February 2023 a qualitative interview study with 10 patients who suffer from chronic IBD. We recruited these patients via the inflammation outpatient clinics of two university hospitals in the North of Germany, contacted them by e-mail or regular mail and informed them about the study with a flyer. Patients usually replied to this email or made an appointment for an interview by telephone. Just before the interview we informed the patients about the aims, benefits, possible risks and the study procedure with an information leaflet and gave them the opportunity to ask questions. All participants provided written informed consent. We conducted eight interviews face-to-face and at the request of the patients, two via video chats. An interview guide based on our previous literature review helped us address all topics of interest. The interviews lasted on average 59 min with a range from 30 to 102 min, were recorded with an audio recorder and transcribed verbatim. Before starting, we received an ethical clearing from the ethics committee of Kiel University.

The interviews were conducted by a social scientist, who is also qualified as a registered nurse, but had no professional experience with IBD. Caring for the patients who participated in the interviews was not part of her responsibilities. The interview guide was also discussed and reviewed with the other scientists involved who have an expertise in philosophy (hermeneutics, phenomenology, ethics), none of whom had professional or personal experience with IBD.

For data analysis we worked with interpretative phenomenological analysis (IPA), a method that is ideally suited for studying people’s lived experience and how they make sense of their experience. IPA has its foundations in phenomenology, hermeneutics, and ideography which entails a focus on the particular. Phenomenology tries to carefully examine humans’ experience and the meaning this experience has for them. For this, researchers have to “bracket” certain questions. This requires researchers to put aside their own views and questions about objective scientific truth in order to get into the experience as it is subjectively given to the subject under study and reported in interviews [34]. However, it cannot entirely be ruled out that a researcher’s personal views on a good life draw attention to certain information in the transcripts during the analysis and thus bias the analysis.

Hermeneutics is defined as the theory of interpretation. It is based on the idea that a part is better understood in its context, the whole, and the whole is better understood when we consider the parts. With a hermeneutic circle, interpretation develops from a pre-understanding or an interpretative question in an iterative process as we go back and forth through the data several times to develop an adequate understanding of it [34]. This is exactly what happens in IPA when we turned to the data in different steps, several times in different ways, passing through the following steps: In a first step, we paraphrased the transcribed data of each individual case in *descriptive notes*, which resulted in a first impression of the patients´ experiences. We worked out linguistic characteristics (*linguistic notes*), which appeared to be very important for our interpretation since meanings were often hidden behind a specific use of language. In addition, we noted questions or ideas about possible interpretations in *conceptual notes* [35]. We conducted this work with MAXQDA, a software for qualitative data analysis and for step 2 we exported the data into an Excel sheet. We developed individual short statements (*experiential statements*) from the notes that represented the experience of the participants. We summarized these statements in a table for each case and then arranged them thematically in *personal experiential themes* (steps 3 and 4). In the last step we presented the resulting themes from the individual cases across cases with anchor examples into *group experiential themes* [35].

Phenomenological studies are only concerned with identifying the core elements of a phenomenon. Thus studies using IPA for analysis usually have small samples [36]. Studies with three, six or even single case studies are not uncommon and are acceptable due to the IPA evaluation guide if the evidence of themes are well reported [37]. The criterion of data saturation, which is used, for example, in grounded theory for theoretical sampling, is not applied here [36], however, it could certainly be argued that sufficient data saturation can improve the reliability of the results. In our study, an inclusion criteria was that participants also had to take part in another study on mobile health technologies [8]. This factor presented an additional challenge for patient recruitment.

## Results

Six female and four male patients took part in the interviews. Patients were on average 31-years-old (range: 19–44) with average of disease onset at age 25 (range: 15–43). Most of them suffered from Crohn’s disease and three from ulcerative colitis. None of these patients already had a stoma. All participants spoke German fluently and only one patient had a migration history. Four patients were in a relationship, but only one of them had children. Two patients were looking for a partner, the other four patients provided no information about their intimate relationships.

The data analysis resulted in five group experiential themes with several group experiential statements that summarize and interpret equal or similar experiences of individual people. Below we report only on three themes (Table 1), since one article would not suffice to cover the richness of the results. These themes provide most significant information for the further development of PRO´s. The other two themes (other people’s responses and strategies to deal with others’ responses) are the subject of a second article with the focus on stigmatization and strategic disclosure which we plan to publish at a later date.

**Table 1:** Group experiential themes and group experiential statements

Group Experiential themes	Group experiential statements (number of patients who mentioned this theme)
1: The body as an obstacle for life options and plans	1a. Abandoning previous life plans [5]
	1b. Renouncing (long) journeys [5]
	1c. Limited recreational opportunities [8]
	1d. Restricting life to the essential requirements and a confined area [6]
	1e. The tendency to social withdrawal [7]
	1 f. The difficulty of building a partnership and family [5]
	1 g. Fears about the future [3]
2: Perception of the body and self	2a. Burdened from a changeable body image [8]
	2b. Burdened from an unfeminine or unmasculine body image and a threatened self [3]
	2c. Perceiving the body or the disease as another subject [7]
	2d. Perceiving oneself as weak [9]
	2e. Perceiving oneself as less worthy [3]
3: Perception of life as good despite suffering	3a. Adjusting expectations for a good life downwards [5]
	3b. Acceptance of disease is key to having a good life with it [3]
	3c. A good life with (deep) happiness [3]

In the following presentation of the themes we follow Smith’s suggestion in his IPA quality evaluation guide to use at least three interview extracts for each statement [37]. The names in the interview quotes are pseudonyms and patients’ comments have been translated into English for this paper.

### The body as an obstacle for life options and plans

The ill body limits the life options and life plans of patients with IBD for many reasons. The symptoms they experience rob the chronically ill patients of life chances. In addition, they themselves abandon life opportunities because of concerns that they will not be able to cope with the demands placed on them.

#### Abandoning previous life plans

For several of our participants the disease represented a significant break in their biography. Professional or private plans had to be abandoned or modified because they were impossible to realize with the diseased body. The abandonment of these plans, however, was not always self-initiated, as in the case of Sophie. She had been struggling for a long time to become a police officer. But government regulations that prohibit people with chronic diseases from working as a public servant in the police force meant the end of this life goal:

The [diagnosis] came on a Tuesday, I remember that. And Wednesday, I should have, I would have gotten the acceptance that I could go to the federal police. (Interviewer, hence “I”: Ah.) And I had been fighting for this for one and a half years with doctors that they would accept me. Because, because of my height they did not want me at first. (I: Yes.) But I could withstand all the physical demands. And then finally, I had the ‘Go,’ but with the diagnosis I was officially unfit for public service again and there’s nothing you can do about it. (Sophie)

Paul, on the other hand, decided on his own that he could no longer participate in sports competitions, even though sports had been a significant and important part of his life. His illness only allows him to participate in training without any great pressure to perform.

Whereby I had already ruled out for *myself* not to take part in these competitions and so on in the long term. (I: Mhm.) Because that is just increased stress that also involves frequent traveling and so on. Then also of course, yes, this competition situation is again something different than going relaxed to training and so. So, I (I: Mhm.) have actually excluded that for a long time now. (Paul)

In the interview with Vera, it becomes clear that the abandonment of the original plan to work professionally as an artist still makes her feel sad and that instead, she had to choose a profession (social worker) where her illness is socially accepted.

So, I do a lot of art (I: Mhm.) like that. There, too, I would have liked to have dared to put myself into it more, somehow. (…) So, yes, so to speak, yes, I think I could have led a much more free-spirited life. (I: Mhm.) And I’m always so sad about it, (I: Mhm.) that I can’t do that. That at the same time I am very, sec-, or my body is very in need of security. (…) And professionally I ended up in an area that has a fully accepting attitude towards various illnesses and so on. And that’s not an issue at all. (Vera)

The fact that Vera addresses this accepting attitude taken within the field of social work indicates that she by no means takes this attitude for granted, which probably results from the fact that she has already had other experiences.

#### Renouncing (long) journeys

Several participants talked about their renouncement of undertaking long journeys or even travelling at all. The reasons why they avoid travelling are manifold. Heather mentions the expectation, that medical care is insufficient in many countries and decided therefore not to study one semester abroad:

Yes, and now, so, there are now also others who are studying with me, who are thinking about whether they will do a semester abroad, or something like that (I: Yes.) and, I thought to myself, that probably won’t work for me to do that. Because there are often countries without such good medical care (I: Ah, okay.) and then I think that’s probably not so perfect, I also need the medicine every six weeks, that could be difficult. (Heather)

Carla avoids travelling on planes and trains because of the uncomfortable toilets, which she does not like:

I think there are people who can do that. I think there are people who think: ‘I don’t care. I’ll get on the plane, I’ll get on the train, (…) I don’t care, I’ll always find a toilet somewhere, then I’ll just go there. And if I don’t find one, then I’ll just go in my pants. Then I’ll wash my clothes and that’s it.’ So, I think there are these people. I think they can live that too. I can’t. (Carla)

Daniel has almost completely renounced travelling, even though he loved doing it:

Travelling is now very difficult or no longer possible. I used to love travelling. (Daniel)

#### Limited recreational opportunities

The fatigue and lack of energy often associated with the disease makes patients less able to take advantage of many recreational opportunities. Daniel presents this very clearly:

Doing sport is also practically impossible. (I: Mhm, what kind of sport did you do?) Mountain biking, running – about at least 10 kilometers, three times a week.” (I: And you don’t do that anymore?) It’s impossible (…) I don’t have the strength for it. (Daniel)

Hannah cancels even everyday meetups with friends or birthday invitations due to lack of energy:

Nowadays, if somehow a gathering is supposed to take place, like for birthdays or similar things. I will sometimes cancel very spontaneously, because if it’s in the evening after a long day at work, (I: Mhm.) then (I: Yes.) I actually just want to (not understandable) no? Just stay at home, have my peace and quiet. (Hannah)

Peter worries that too many leisure activities could trigger stress, which would have a negative impact on his disease:

Oh yes, well I’m still in the theater group. Acting is a lot of fun. I’ve been doing it since I was a child. (I: Yes.) So. And as you can hear, many, (laughs) many things are now coming together again. (I: That sounds like a lot, yes. (laughs)) (…) There is also fear. About whether I’ll overdo it again, have too much stress. And that has an effect on the disease again. (I: Hm. Yes.) It just always resonates again somehow. (Peter)

In these interview excerpts, it becomes clear that patients are limited socially, spatially, and in terms of the types of living options available to them. The restriction of life becomes even more clear with the next experiential statement.

#### Restricting life to the essential requirements and a confined area

Especially during disease flare-ups, but also in between, some patients feel that their lives are extremely restricted, reduced to the bare necessities like work, sleep and some housework and confined to a limited area. Hannah reported:

All I really do now is work and just about manage my household. And that’s it. (I: Yes.) That’s how I perceive it. (Hannah)

Carla has even already decided that she will never relocate from her current living environment and has secured this future for herself by purchasing a house in her city.

Yes, I have already built myself such a, my nest. And I bought a small townhouse two years ago. And I have also decided that I will not move away from the city again. I have my doctors here, I am, um, well-networked here. I have my environment here. And that’s important for me and my health. (Carla)

During flare-ups, Daniel hardly dared to go out of the house fearing the symptoms.

Especially in the flare-ups it was so bad, there was one day when I could go out, I could go for a walk for five minutes. I also managed to go shopping. But how many days were there where that wasn’t possible? (I: Mhm.) Where I then maybe didn’t dare at all, because it then started again with the stomach and everything. (Daniel)

An interesting point in the interview with Carla was the information that she appreciates pure nature as a place where she feels safe and comfortable. While hiking with her parents in the mountains, she felt free from societal observations and was not afraid or ashamed of other people’s gazes:

Because when you go hiking in South Tyrol- Well, I was with my parents. And that’s in any case not so bad. Um, you’re in the middle of nature. So, when I had to go to the bathroom, I said: “You go ahead. I’m faster anyway. There was no one there. Then I went to the next tree or something. I squatted down there. I found that somehow totally natural and somehow beautiful. (Carla)

While Carla is often afraid that in quiet situations someone might hear her bowel sounds, nature becomes in this way a safe space where no one can embarrass her.

Retreating to a safe place and limiting activities to the bare minimum is also associated with social withdrawal, an experience that most respondents talked about.

#### The tendency to social withdrawal

Several interview quotes indicate that respondents tend to withdraw socially and also accepted this disengagement. Daniel, however, described this as a burden:

I’m not as socially active as I once was. (I: Mhm.) That really gets me down. (Daniel)

The reason for the social withdrawal is partly a conscious decision, for example having to continuously meet the special needs of the diseased body. Tom, for example, made a conscious decision not to share an apartment with another student because he has special hygiene requirements in the bathroom and kitchen. Instead, he decided to live on his own.

That was also one of the reasons why I said I wanted to live alone. Yes, (I: Yes.) because sharing an apartment was somehow not my thing. (I: Yes, yes.) Above all I am, um, what I learned with the colitis, was um, to be very hygienic and very orderly, (I: Yes.) when it comes to the bathroom and kitchen, for example. (…) It’s always been very important to me that the bathroom is very clean, (I: Yes.) Because I spend, I usually spend more time than other people do, and then I want, (I: Yes.) to be clean. (Tom)

Suffering from symptoms also leads simply to a lack of the willingness to meet other people:

It’s then of course sometimes true that when I’m not doing so well, I don’t necessarily want to meet up with friends or anything right then.” (Heather).

But not only the withdrawal from the circle of acquaintances, friends, and workplace is associated with the disease. The development of close social ties with a partner and building a family are also associated with difficulties.

#### The difficulty of building a partnership and family

In the interviews, some patients talked about their difficulties in establishing and maintaining a partnership. The symptoms of the disease are an obstacle especially in the initial phase of a love relationship.

And for me, dating is extremely difficult. Because I wouldn’t sleep over at someone’s house just like that. Because I wouldn’t want someone spending the night at my place on the spur of the moment, either. Because at some point the TV is off, the silence is there. And what if I have to go to the bathroom? Or if my stomach starts grumbling? That’s the horror for me. Yes (…) Yes, I am single. And, um, it, yeah it’s just difficult because of that. (Carla)

Peter fears that he will not be able to maintain the relationship with his girlfriend because he tends to withdraw from her when his condition worsens.

I just don’t know whether when I feel worse again, I’ll try once more to push my girlfriend away from me. (I: Hm.) And close myself off again and the like. That then maybe an actually good relationship can fall apart. (Peter)

For various reasons, respondents also see parenthood as problematic. One patient is concerned that any child she had would inherit the disease, another is afraid of not being able to care for the child sufficiently.

(…) if there is a risk of me passing it on, no matter how big or small that risk is, I am currently at the point of saying, I don’t want to bequeath that. (Anne)

After lengthy discussions, Paul and his wife decided to have a third child, but his feelings about this are mixed between joy and fear of the multiple challenges associated with parenting:

Well, it’s like this now, we’re having a third child. (I: Ah, okay. Fine.) That was so to say also a long decision-making process. That’s the one thing. But I’m also a bit jittery. Yet, I am also very much looking forward to it. But as I said, I also have a lot of respect for myself again now, though it’s actually clear that my wife knows that there’s more on her plate now. (Paul)

Daniel, who also suffers from an additional disease, is afraid that if he adopts a child, he will not be there for the child until it grows up.

If I adopted a child now, then I also want to somehow be there for the child, at least until it is all grown up (I: Yes.) And I can’t guarantee that. So, I decided against it. (Daniel)

#### Fears about the future

Three patients talked about their fears regarding the future that are related on the one hand to the feeling of not being able to meet future professional demands, and on the other hand to possible surgical interventions resulting in a life with a stoma.

What will the future look like? What else will I have to face? Will I need an artificial bowel outlet at some point? Will I even be able to finish my degree? Will I be able to find a job where I can reasonably juggle my doctor’s appointments and everything? So, those are a lot of fears about the future. (Anne)

Daniel has already met several patients with a stoma and as a consequence, he fears that this will be the case for him as well.

I’m just a little afraid that at some point, things won’t go on like they are now. Because I have already met many patients. Most of them have a stoma or an artificial bowel outlet. (I: Yes.) I am a bit afraid of that. (Daniel)

Professional requirements put pressure on Peter and make him doubt whether he can meet these expectations.

Well in the times before the infusion, I already had, I don’t want to say now existential fears. But I have always had fears. (I: Hm.) Will I be accepted in my job? Can I perform well enough in order to get ahead? Or am I hanging around somewhere where I don’t even want to remain? Because I simply *can´t* deliver the performance anymore? So, the fears, I already have them. (Peter)

In summary, it becomes clear that IBD significantly affects patients’ life opportunities and that especially things like home, family, work and friendship that contribute to deep happiness in the sense of Foot [10], are more difficult to achieve or to maintain.

### Perception of body and self

In this section we will show that the changes in body image due to the disease and therapy are a burden for patients. The body, which is difficult or impossible to control, leads some patients to perceive this body as an “other” with whom they have to fight a battle. The intrinsic sense of weakness and the reactions of other people gives them an image of themselves as weak and lowers their self-esteem.

#### Burdened from a changeable body image

Several patients shared about how their body image has changed as a result of severe weight loss during flare-ups or weight gain due to cortisone therapy. The associated distress is described as being scared of one’s own body, insecurity and loss of confidence, or distortion of body image. For example, Sophie described:

And recently I found old pictures where you could see a comparison to now and I was very shocked. (…) I was very, very underweight. Weighed between 41 and 43 kg. Um, that wasn’t a pretty sight, but I realize that I still partially have such a distorted image, because my weight has really only remained stable for about half a year, and before that it was fluctuating. (Sophie)

For Vera, the weight changes represent a loss of confidence in her own body, which also manifests itself as insecurity in her own self:

Then the body changed in such a way that I put on a lot of weight again incredibly quickly due to the cortisone therapy. (…) because I could trust so little in my body and also because I am a bit vain. Um, because I was constantly somehow getting this cortisone bomb once a year. So I looked completely different every year. So, um here too, there is just no having confidence. So, well there is just such a great uncertainty, um with myself. (Vera)

The severity of the burden of weight change was evident in the interview with Anne, who describes her reaction to looking at a photo of herself as like having a nervous breakdown:

And had a meltdown when I saw the pictures. Because I was just *so fat* in my eyes. Even though I knew it was basically from the cortisone. (Anne)

#### Burdened from an unfeminine or unmasculine body image and a threatened self

Due to weight loss, some patients experience the appearance of their body as being incongruent with their own body norm as part of a specific gender role such as “an adult woman” or “father of a family.” Carla, for example, feared being perceived as looking like a girl, something that she felt uncomfortable with:

But at some point, um, of course this femininity just fades. And it had always been important to me that I also feel a bit feminine. And if there’s no proper bottom or proper breasts or so that are really apparent, then, um-. (…) Yes, I didn’t want to look so much like a girl, but I wanted to be perceived as a woman. (…) when I noticed that it was so little, that it was decreasing, that, um-. Yes, of course I didn’t feel comfortable with it.” (Carla).

Paul regrets the muscular degradation of his body, which leads to significant loss of physical strength and limits him in his role as a family man who is active in sports with his sons. This threat to his self is difficult for him to cope with:

So, the worst thing for me, to say, is just this whole muscle deterioration. This, this weight loss. (…) And that is what is also well, emotionally difficult. (I: Mhm.) That you just have one such, I’m exaggerating now, but sometimes, I only feel like half a man. (…) Yes, especially in the role as a family man (I: Mhm.) um, it’s quite difficult. I have two sons. (I: Yes.) They are also at about that age now. One is nine, the other will soon be seven. And um, yes, it’s no longer for it to be fair. Other families can go just go off to the [Mountains] or do this or that activity (I: Mhm.) For me, that’s always a challenge. (Paul)

Another patient talked about the time when she was in her adolescence, a time when the disease challenged her identity as a teenager, as she could not party like her peers. In the interview, she made it clear how much she would have liked to see herself as someone else:

Yes, because I was actually already so-. Well, I’ve always been a person who likes being around other people and doing a lot. And somehow also whoop it up sometimes. And that just wasn’t possible. (I: Yes, yes. So that limited you in your life, didn’t it?) Yes, in being young. So-. (I: In being young? Yes.) Yes. So now I don’t mind not being able to drink alcohol, but back then it was (laughing) really somehow- (…) Yes. Yes, and I would have liked to have seen myself *differently*. So, I would have liked to have seen myself being much *more* right in the middle of things (laughing) than I already was then anyway. Yes. (Vera)

#### Perceiving the body or the disease as an “other”

In several interviews it became apparent that the patients speak about their body as if it were not a part of themselves. The body appeared almost like another person that speaks with them, limits their freedom, and at which they could be angry.

I’m always rather a bit tense, which has to do with the illness. Will that work for me right now, and so on. Um, or is my body going to thwart my plans (…) Yes, I would like to, I would like to be able to do more, more sometimes. (I: Mhm.) So, the, the will is there. But just the body (I: Mhm.) tells me then-. (Paul)

In the conversation with Anne, it seems as if she and her body are two different subjects who have to negotiate something:

And like that, I’m trying to find a way so that to some extent, me and my body are in agreement. (Anne)

For Heather, the body is someone she is quite angry about:

Yes, so partly, yes, I’ve already noticed like that, I’m already a little angry at my body (I: Angry?), yes, because it’s doing this to me and I’ve actually always eaten a relatively healthy diet, I don’t drink alcohol, I don’t smoke, actually I’ve paid attention to my health and then I get something like this. (Heather)

For some patients, however, it is not the body that is the other, but the disease that is in the body:

I do look at, or overthink what I’ve been eating. What might Crohn’s disease not have liked, for example, something too spicy, too greasy? Mhm, or maybe I did go to parties too much, or maybe the alcohol consumption was too high or something. (Sophie)

Hannah also refers to her disease as someone else, as a roommate to whom she has given a first name and from whom she obviously distances herself. It seems as if she always has to fight a battle with this other, in which sometimes she wins, sometimes the other.

Yes, the roommate is stronger, right? (I: Your roommate? Mhm.) Yes, exactly. I also named him Horst. So when I say: “Horst is getting on my nerves today,” then Crohn is just a bit stronger. Then he has the upper hand. (I: Mhm.) But I don’t let him have it so often, right? (…) because I don’t know how Horst will react to it, OK? (Hannah)

The ways we can interpret this attitude to the body, self and disease appear ambiguous. Is the distancing from the body merely an expression of an internalized Cartesian dualism of mind and body to the point that it is evident in the patients’ everyday language [38]? Or can we interpret this distancing as alienation from the body, as it is also evident in other diseases [39]? And what does that mean for the practice of clinical care? We will come back to these questions in the discussion section.

#### Perceiving oneself as weak

In the interviews, we asked patients what adjectives they would use to describe their bodies. In addition to a few positive attributes, a large number of negative ones emerged. One of the most frequent adjectives was the word “weak,” but the terms “less muscular” or “drained” also appeared repeatedly.

And because of that, I always feel that I am sometimes weak. Not as much, not as resilient as others. (Carla)

Peter mentioned also the expression “to be broken”:

Well during a flare-up, I feel very, very broken. Or-. (I: Hm.) So my body feels very broken, very limp. And I say, um very drained. So the energy was just gone. (Peter)

Because Tom has often heard during medical consultations that his intestinal walls are porous, he has also incorporated this image of fragility into his body image:

Yes, weak, weak and um, fragile. (…) And porous, porous is also so- porous is such a nice word. (I: Porous.) Porous. (I: Yes.) Because I always, um, connect that when I have a relapse, it’s quite often said that the intestinal walls are porous. (Tom)

#### Perceiving oneself as less worthy

The difficulty of meeting the expectations of society in their professional and private spheres combined with the associated social withdrawal makes some patients feel that they are of lesser worth, and that their self-esteem has declined.

It’s a bit like a decline in value. Well also, the self-esteem, that has already decreased. (…) I think I would be a bit more self-confident if I didn’t have the disease. (…) I have already caught myself thinking that maybe I’m less lovable because I’m ill. Well, that’s probably complete nonsense, but every now and then things pop up and I discard them again. But I also think to myself, such nonsense, not true at all. But, yes, it-, the thoughts come naturally. (Carla)

Daniel explains the decline in his sense of self-worth with the fact that he himself feels a bit dirty:

Well, before I definitely had more self-confidence than I do now, (I: Yes.) after the disease. (I: But your self-confidence has suffered?) B: Yes, of course. You always feel like you are a bit *dirty.* (Daniel)

Peter also attributes the perceived loss of self-worth to the fact that he carries a stigma with the disease:

So, before the infusion, I already felt - well worthless sounds too harsh. (I: Hm.) But already impaired, worth less. (I: Hm.) That one has such a stigma about oneself. (Peter)

When summarizing patients´ perception of their body and self, it seems that some patients do not feel at home in their own body, as its appearance often changes, appears unfeminine or unmasculine. This sometimes results in a distorted image, which makes some patients feel insecure in their being. Additionally, some patients perceive their body as being an “other.” Perhaps the uncontrollability of the processes in their body makes them feel alienated from it. They perceive this body as someone else, who has their own will and with whom they have to fight a battle. Further, the physical weakness resulting from the disease makes them feel weak and less worthy because they carry a stigma and are insecure about being able to adequately fulfill future demands in their private and professional lives.

### Perception of life as good despite suffering

When considering the patients’ limitations in making use of the full range of life options and plans, we asked them in the interviews whether they perceive their life as being good. The answers were surprising. Almost all respondents mentioned their life as being good despite their suffering, mainly because of their deep happiness with family, friends, and work or because of their being able to cope well with the disease. However, patients´ concepts of a good life partially differ from Foot’s concept. From their answers it seems that they have lower expectations for a good life, or that they have already reduced their expectations due to their suffering.

#### Adjusting expectations for a good life downwards

For some respondents, having a home, living in a free country, being financially secure or simply leading a quiet life is enough to make them feel they have a good life:

I would definitely say I can lead a good life. (I: Mhm.) Yes, so that uh-, I have a roof over my head et cetera, right? (I: Mhm.) Well, I’m just not somehow-, my existence is not threatened. (I: Mhm. Yes.) And accordingly, I would definitely answer the question with yes. (I: Yes.) (Vera).

And I don’t really have such big goals. I just want to have a quiet life. Just a balance between being financially secure, but still somehow having the freedom to say, no, I’m on vacation here, I’m going away here and there. (…) That’s why the only goals I have are simply to live the day and fall into bed at the end and be satisfied. And to stop complaining about the day. (Sophie)

Here, I feel like I’m in a free country. So, I have the feeling I have all possibilities. (Carla)

#### Acceptance of disease is key to having a good life with it

For Sophie, just being able to cope well with the disease and accept it is essential for a good life:

If you feel comfortable and can deal with it, it is already a good life. And I have found my way of how I can deal with it, how I can satisfy, let’s say the people close to me. So that they don’t totally worry or despair. That’s why I would say that a good life only begins when you find your own way of dealing with it, that you accept it above all. Because acceptance is the greatest thing, I think, in the story. Because if you don’t accept it and don’t want to admit it, I think you won’t be satisfied and above all you won’t be satisfied with yourself. (I: Yes.) and that’s why I would say I lead a good life. (Sophie)

Tom and Anne also share this view of the importance of accepting their illness:

(I: To what extent can you live a good life with your illness?) Yes, if you accept it, adjust to it and, uh, accept and learn. Learn a lot, learn more and get to know your own body and also be aware that body and mind are always connected, um, then you can live very well with it. (Tom)

But yes, I think once you have accepted everything and have good therapy, you can definitely lead a good life. (Anne)

#### A good life with (deep) happiness

Carla and Peter define a good life with the disease in a way that is very close to Foot´s notion of deep happiness:

What is a good life? Yes, yes I think-, I think that, that I can do that already. Um, a good life for me is having a meaningful job. Um, a positive way of being together in the family. Social relationships, that’s clear for me. Um, also a, a, so a balance between freedom and obligations. And that also results a bit from work and the environment. And what you create for yourself. (Carla)

But if I have a job, a profession that’s fun and fulfills me, that is very, very important. (I: Hm.) If I have friends. If I’m in a good relationship. So, if all these basic conditions are good. If I can also now eat in the sense of the illness, just what I *feel like* eating. If I can do what *I want* to do. (I: Hm.) And am not restricted because of these factors, which *I can’t influence*, (I: Yes.) I would say, I am satisfied. (I: Hm.) And I can then also lead a good life. (Peter.)

For Tom, it is not *deep* happiness, what he needs for a good life, but it includes activities that make him happy, such as going to a concert or being active in sports:

(I: What do you think is part of a good life?) For example, um, going to concerts, uh, going to festivals, um, being active in sports. Um, I’ve had to *learn* a bit in the last few years to become a bit more spontaneous again, because my life is very regimented for me. Because then, of course, it’s just easier for me to live with the illness. (I: Yes.) Um, that’s why I’m a very-, yes, or have lost a lot of spontaneity. And I’ve now, but actually in the last year, I’ve got that back a bit. (Tom)

## Discussion

Many of our findings in this study have also been identified in other studies. For example, Fourie et al. (2018) developed in their review of 23 qualitative research studies about living with IBD five main themes and several subthemes. The main themes were: “Living with exhaustion”; “Living in a flawed body”; “Living in secrecy”; “Living with restriction;” and “Living with fear.” An overarching theme also emerged: “Living in isolation and exclusion” [40]. These themes and subthemes are, despite their different formulation, very similar to our findings described above in the subsection *The body as an obstacle for life options and plans.* However, our findings regarding the patients´ *perception of the body and self* are only marginally developed in previous publications. Fourie’s study also revealed a “damaged body image” [40] and Ruan et al. (2020) mentioned “the body having flaws” [33].” However, research on the accompanying “ontological insecurity” [41]^1^ has been lacking, In our study, Sophie described this as a “distorted body image” and Vera as “severe insecurity with myself.” This insecurity may also be exacerbated by the fact that weight loss can cause the body to lose visible sex-specific features, such as breasts and buttocks in women and muscles in men, making patients feel compromised in performing their gender roles and unattractive for potential partners. These fears of being perceived as unfeminine or unmanly have already been investigated with one item as part of the Hopwood Body Image Scale in a quantitative study about body image dissatisfaction in IBD, however, the study revealed no results for this specific item. [42, 43]. Because females and young patients tended to have higher body image dissatisfaction scores in this study [43], one can assume, that in these groups the body image is of high importance, especially as younger patients may still be looking for a partner and want to start a family. Nevertheless, regardless of the person’s biological sex, the burden of a changeable, unfeminine or unmasculine body image should be given more attention in clinical practice, as dissatisfaction with the body is often accompanied by psychological distress [13] that requires care. It is precisely this care that is often lacking and not part of usual care for patients with IBD.

As we have reported in the results section, when patients talk about their body or the disease, they sometimes take a third person-perspective and tend to describe their body as being someone else. One can assume that this use of language is simply an expression of the Cartesian dualism of the mind and body still common in everyday language [38] and health care. The continuing organization of medicine into organ-specific departments and the intensive research about the molecular causes of disease supports this way of thinking, although mental illnesses do find their expression in the body and conversely. And perhaps our questions in the interview could have evoked this dualism, too, when we asked patients about adjectives to describe their body. One reason why we think that this is not the whole explanation is the fact that patients talked about their body or the disease as if this is somebody who *acts* on their own and who can hardly be influenced by the patient´s will. The body is out of their control, and this is a characteristic which we usually attribute to another person. In this way the body or the disease itself becomes an “other” or “alien”, a phenomenon which is also evident in other diseases [39] and the self is “disembodied” [41]. Laing illustratively described the position of the disembodied self: “Instead of being the core of his true self, the body is felt as the core of a false self, which a detached, disembodied, inner, true self looks on at with tenderness, amusement, or hatred as the case may be” [41, p. 69]. Disembodiment leads the self to take an observer perspective on the body. Some authors have interpreted this as an avoidance strategy, which emerges when patients feel their body being observed by others [44]. This explanation seems to also be plausible in the case of IBD, since patients are at risk of being stigmatized and they sometimes feel ashamed about their bodily behavior. Another explanation is, that patients cannot be accountable for the disease, when another person, namely the disease called by a first name, is the cause for their symptoms [39]. In this sense, the alienation from the body or from the disease represents coping strategies. The question is, which implications should that have for clinical care?

If we adopt an ethics of care perspective that emphasizes the interaction with patients [45–47] and focuses on the understanding of the particular situation of the patient [48], then our results have several implication for clinical practice. First of all, health care professionals should realize this alienation from the body and work out potential care needs by investigating the meaning this alienation has for patients. Until now it seems unclear which function(s) this alienation has for patients with IBD. Do they benefit from this strategy—and if so, how? Or does it impact them negatively? To answer these questions, further research is needed which includes the development of assessment scales for the detection of bodily alienation and its meaning for patients. Secondly, the alienation from the body should be empathically understand and accompanied by health care professionals so as to alleviate patient suffering [39]. Here, key is recognizing that this suffering not only results from the symptoms, but from patient’s fear about the societal responses to their “dirty, smelly or noisy” disease and body.

Many interviews show that suffering from IBD is often a result from living in a space where societal norms limit the life of patients. One is reminded of Sartre´s statement in his famous play *No exit*: “Hell, is other people” [49]. But how can patients get out of this hell? Withdrawing from society to a confined, safe area cannot be the only solution. Such a societal withdrawal may result in unwanted solitude that in turn, could impact mental health. An alternative solution may be to become independent from the judgement of others and from harmful relationships. But the task of engaging only in relationships in which the person with IBD is appreciated and emancipating themselves from the internalized norms by regaining a self-confident “self” is not an easy undertaking. Therefore, we suggest that patients need psychological support which should ideally be offered at an inflammation outpatient clinic as part of usual care. Furthermore, societal awareness about chronic diseases must be strengthened in order to sensibilize people to the needs of patients with chronic diseases such as IBDs and to prevent the stigmatization of these patients. For this, patient narratives are important and should be used in public health education campaigns from national centers for health education (e.g. the German Federal Centre for Health Education) or, as another possibility, in school education. If people learned more about the etiology and burden of inflammatory diseases such as IBDs, they would better understand those affected. Furthermore, this could lead others to adjust their own lifestyles (as this has an important influence on the onset of inflammatory diseases) and thereby prevent their own suffering from disease in future. Moreover, patients could expect to receive more understanding in their social environments.

Looking at Foot´s concept of a good life and the notion of “deep happiness” which is something more than just joy or contentment, our results support the claim that for a good life in the patients’ own view, a deeper sense of happiness is essential. For deep happiness it seems that a few basic things are primarily necessary: home, family, work, and friendship. According to Foot (and our interviewees), virtuousness is conducive to a good life and suffering is not an obstacle [10]. It was perhaps not surprising that most of our patients in this study, when asked about their quality of life found their lives to be good despite their suffering. However, Foot´s concept of the good life entails more dimensions than deep happiness. Flourishing and the realization of human faculties [10] could, from the first view, only partly be realized by the patients in our study. Some patients had to abandon previous life plans and adapt their realization of capabilities to the possibilities of the disease. But it seems that at least some of them have been very successful in this adaptation and that they are discovering new ways of human flourishing for themselves, so that they no longer suffer from their limitations. This can give hope to other patients, who have not yet reached this point, but might also arrive there someday. Nevertheless, even when patients cope well with the disease, living with a chronic disease like IBD requires from them constant attention and the ability to adapt, particularly when living conditions have changed, which sometimes occurs during the course of life. A good life for a patient affected by IBD, or another chronic disease, may be rather a fluid idea that is under constant construction and reconstruction by the patients themselves. It is not a fixed idea that would lead to a set of objective evaluation criteria. The concept of a good life is therefore an evaluative concept that people are working on in the context of the changing conditions of their embodiment and in the different contexts of their life world. This fluidity of evaluation of lives as good, better or worse, and patients’ efforts in working conceptually on the ideals they have in their lives would be essential to include in patient reported outcome assessments.

## Conclusions

As part of precision medicine, we think that health care professionals should monitor the impact of therapeutic interventions on patients` opportunities for a good life beyond HRQoL. This would entail recognizing patients´ own life goals and plans, including patients’ own readjustments to their perceived opportunities under the conditions of IBD, and their ability or inability to reach these goals and their adaption to their feasible options. Changes in body image and the associated impact on self-esteem should be regularly assessed and supportive psychotherapy offered. The Body Appreciation Scale [50] could be used for this purpose or existing PRO scales for IBD could be improved. Since a family is an important source of deep happiness for most people, education on sexual dysfunction, family planning, and pregnancy should also be given more attention in health care practice [51].

This study shows that Foot’s multidimensional concept of a good life is well suited as a basis for investigating the effects of PM on the lives of people with IBD, since interviews based on this concept produce results that go beyond the understanding of HRQoL. In particular, the body as an “other” is a finding which was not revealed in other studies on HRQoL in IBD. Comparable studies on living with other inflammatory diseases should follow to further demonstrate the advantages of our methodology.

### Limitations

Our study can only provide some ideas for the further development of PROs in IBD. Additional quantitative studies should test our results. The extension and improvement of existing PRO scales should also be re-examined for validity and reliability and this requires further research.

### Electronic supplementary material

Below is the link to the electronic supplementary material.

Supplementary Material 1

## Data Availability

The raw de-identified data may be made available upon reasonable request from the corresponding author, and after explicit permission of the study participants.

## Footnotes

1 As Ronald D. Laing we use the term “ontological” as an adjective in order to describe the insecurity of being. However, comparing with the work of Laing, the ontological insecurity our patients´ experience was not accompanied by severe psychiatric disorders.

## Abbreviations

IBD: inflammatory bowel disease
CD: Crohn´s disease
UC: ulcerative colitis
QoL: quality of life
PM: precision medicine (or: personalized medicine)
PRO: patient reported outcome
HRQoL: health-related quality of life
