# Supplementary material for: The body as an obstacle and the “other”. How patients with chronic inflammatory bowel diseases view their body, self and the good life
Source: BMC Med Ethics. 2024 Jul 24;25:82. doi: 10.1186/s12910-024-01076-2 (PMC11267929; doi:10.1186/s12910-024-01076-2)
Supplement: Supplementary file 1 — Supplementary Material 1 [file 12910_2024_1076_MOESM1_ESM.docx]

**Interview guide to body experience, self-identity, (self-)stigmatization, mHealth and living well with CID.**

**Introduction**

- Brief introduction of the research project and objectives, any comprehension questions about the project.
- Everyday language introduction to the specific interview format (interviewer more of a listening role; interviewee's personal perspective of interest)
- Information, data protection, confidential treatment of data (pseudonymization, no disclosure to third parties)
- Short questionnaire
- Check willingness to be recorded, explain reasons if necessary
- Switch on recording device

**Body experience/body image**:

1. First of all, I am interested in how you experience your body, its function and your physical appearance differently today than before the disease? Would you share with me something about this?

What thoughts and feelings has this change triggered in you?

How have you dealt with this change?

If you were to describe your body with adjectives (describing words), which adjectives would you choose? (Why? And during a flare-up?)

**Identity (Self):**

2. How does the disease affect your image of yourself as a whole person? (Or asked another way, if you were to describe yourself, who were you before the illness and who are you now?)

How has your image of yourself changed? (Just by the disease, by other people, by therapy?)

**Stigmatization:**

3. Who have you told about disease? (Why this particular person? On what occasion? Was the disclosure planned?)

How did the person/ people react to the information? (How do you explain this reaction?)

What thoughts or feelings did this reaction trigger in you? (Did the reaction of others change your image of yourself? In what way?)

Who have you concealed your illness from and why?

Under what circumstances would you disclose your illness to these people? (What opportunity would you choose to do this?)

**Life goals, life opportunities:**

4. How do these changes affect your life, life opportunities, and life goals?

To what do you attribute the limitations in your life? (To the disease only? To the stigma from other people? To your own thoughts and feelings? What do these limitations mean to you?)

To what extent can you live a good life with your disease? (What is part of a good life for you?)
